# Systematic review of patient safety incident reporting practices in maternity care

**DOI:** 10.1136/bmjoq-2025-003432

**Published:** 2025-10-05

**Authors:** Emma Beecham, Gráinne Brady, Syka Iqbal, Qanita Fatima, Saeeda Arshad, Paulina Bondaronek, James O’Carroll, Stephanie Glaser, Dimitrios Siassakos, Katie Gilchrist, Jenny Dorey, Rebecca Knagg, Cecilia Vindrola

**Affiliations:** 1 Department of Targeted Intervention, University College London, London, UK; 2 Department of Psychology, University of Bradford, Bradford, UK; 3 Institute of Health Informatics, University College London, London, UK; 4 University College London Hospitals NHS Foundation Trust, London, UK; 5 Institute for Women's Health, University College London, London, UK

**Keywords:** Obstetrics and gynecology, Patient safety, Systematic Review, Incident reporting

## Abstract

**Problem:**

Patient safety incident reporting in maternity care is central for improving safety, yet inconsistencies in reporting practices and limited understanding of system functionalities may reduce its effectiveness.

**Background:**

Reporting incidents allows healthcare providers to identify safety issues and implement improvements. However, variations in reporting practices, particularly in maternity care, have been found across different healthcare settings. Despite the growing use of electronic systems, challenges such as under-reporting, lack of feedback and insufficient organisational learning persist.

**Aim:**

This review explores how patient safety incidents are reported in maternity care, identifies the systems used globally, examines potential barriers and enablers to reporting, and highlights gaps in existing research and practice.

**Methods:**

A systematic review was conducted, analysing studies that focused on incident reporting practices in maternity care. An artificial intelligence text analysis tool (Caplena) was used to aid the synthesis of the study data. Methodologies included quantitative surveys, qualitative interviews and mixed methods approaches.

**Findings:**

A total of 15 studies from seven different countries were analysed. Reporting systems ranged from traditional paper-based methods to electronic platforms. Barriers included organisational culture, time pressures and inadequate reporting platforms. Enablers involved supportive leadership, training and user-friendly reporting systems. Substantial gaps included the under-reporting of near misses, lack of feedback mechanisms and insufficient attention to staff experiences.

**Discussion:**

The findings highlight the need for consistent, user-friendly reporting systems and fostering a supportive, non-punitive culture. Strengthening and improving feedback mechanisms is also critical to enhance reporting practices. Recommendations are provided for designing future reporting systems.

**Conclusion:**

Improving patient safety incident reporting in maternity care requires system improvements, cultural changes and further research to address identified gaps and optimise incident management systems.

**WHAT IS ALREADY KNOWN ON THIS TOPIC**

- Patient safety incident reporting in maternity care is recognised as crucial for improving safety in maternity care, however, there are inconsistent reporting practices across different healthcare settings.

**WHAT THIS STUDY ADDS**

- Significant gaps in current practices include the under-reporting of near misses, lack of effective feedback and insufficient consideration of staff experiences.

**HOW THIS STUDY MIGHT AFFECT RESEARCH, PRACTICE OR POLICY**

- This study highlights the need for standardised reporting practices, integration of qualitative data into reports and the promotion of a culture of transparency to enhance incident reporting and patient safety in maternity care.

## Introduction

 Patient safety is a fundamental principle of medicine and healthcare, which encompasses a framework of organised activities, processes, technologies, actions or omissions that result in hazardous, dangerous conditions and/or cause unintended, avoidable harm.^1^ The reduction of maternal mortality is a key global health priority and target of the WHO’s Sustainable Development Goals.^2^ In the UK, studies have highlighted the urgent need for enhanced risk management in maternity services.^3^,^5^ The National State of Patient Safety report^5^ indicates that the rates of stillbirths, neonatal deaths and maternal deaths have worsened in the UK, with maternal deaths per 100 000 maternities increased from 9.71 in 2022 to 13.41 in 2023, calling for systemic change and action. Unsafe maternity care incurs financial and economic costs, additional interventions and consuming resources to manage the immeasurable consequences of patient harm.^2^

Incident reporting is a crucial component of patient safety, serving as a mechanism to identify risks, analyse adverse events and implement preventative strategies. In England, the National Patient Safety Agency^6^ previously outlined a list of maternity-related events that warranted reporting to trigger reviews. This framework has since been replaced by the Patient Safety Incident Response Framework (PSIRF),^7^ which aims to strengthen the reporting and learning culture in maternity services. Several countries have developed patient safety incident response frameworks similar to PSIRF to enhance transparency and learning from adverse events in healthcare. For example, the National Safety and Quality Health Service^8^ Standards in Australia and the Patient Safety and Quality Improvement Act, which established Patient Safety Organisations^9^ and the Network of Patient Safety Databases^9^ in the USA. These frameworks, like PSIRF, aim to shift the focus from blame to learning, fostering a culture of continuous improvement in patient safety across healthcare systems worldwide. For the sake of transparency, and given the global scope of this paper, we adopt the WHO definitions^10^ to ensure consistency in terminology within the context of healthcare. WHO defines an incident as any deviation from usual medical care that either causes an injury to the patient or poses a risk of harm, including errors, preventable adverse events and hazards. An adverse event, according to WHO, is an incident that results in preventable harm to a patient. Additionally, a near miss describes an incident that did not reach the patient. By applying WHO’s internationally recognised classifications, this paper ensures alignment with global patient safety frameworks and facilitates broader applicability across healthcare.

A key question in maternity care risk management is how incidents are documented and analysed to improve patient outcomes. Previous research has examined patient safety reporting practices, identifying essential elements for improvement. Gong *et al*^1^ conducted a review of patient safety reporting systems, identifying potential gaps in system design and proposing strategies for enhancing reporting processes. One critical recommendation was enabling staff to access reviewer feedback, promoting learning and continuous improvement. Transparency in reporting practices is vital to ensure the safety of both mother and newborn, yet there is limited research on the specific factors influencing reporting behaviours among maternity care professionals.

The WHO^2^ recommends implementing reporting systems that clearly define incidents, provide staff training and complement other reports and improvement initiatives. Although there has been advancement in patient safety through strategic planning for health systems, the integration and opportunities to learn from patient safety reports have been slow due to organisational cultures of blame and retribution of those who make errors.^2^

Despite the importance of transparent reporting practices in cases of safety incidents in maternity care, there is limited evidence on the reporting practices used by staff and the factors that promote and hinder reporting. Transparency is critical to safeguard the lives of both the mother and newborn, however, there is little evidence on the reporting practices used by staff.

## Objectives

This review aims to explore how maternity service staff report incidents, the reporting systems that are currently used globally, the factors that act as barriers and enablers in incident reporting, gaps in published research, and recommendations for improving incident reporting and future research.

The review was guided by the following questions:

1. What are the current patient safety incident reporting practices in maternity care?
2. How do healthcare staff currently report incidents?
3. What factors act as barriers and enablers to incident reporting?
4. What are the current gaps in research on patient safety incident reporting practices?
5. What recommendations exist for improving patient safety incident reporting?

## Methods

**Figure 1:**
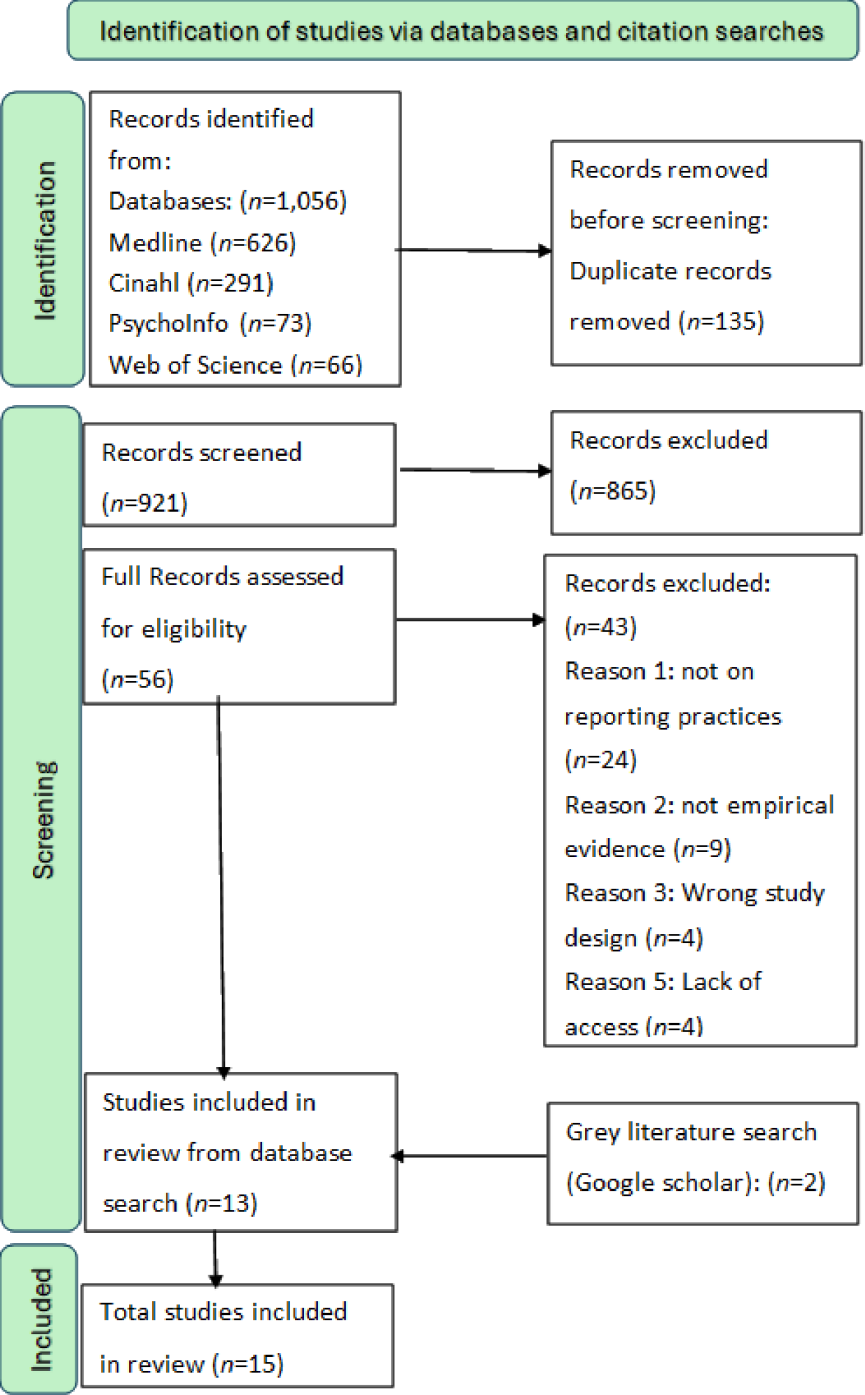
Preferred Reporting Items for Systematic Reviews and Meta-Analysis flowchart of the literature search and selection of studies.

A systematic review was conducted on published literature from database inception to 25 June 2024. The review followed the Preferred Reporting Items for Systematic Reviews and Meta-Analysis statement to guide the reporting of the methods and findings, see figure 1.^11^ The review was prospectively registered with the International Prospective Register of Systematic Reviews PROSPERO (CRD42024547620) on 25 June 2024.

### Search strategy

The search strategy was developed in collaboration with a university librarian (WH), piloted in Medline and revised by two researchers (EB and CV). The final search strategy was conducted in four databases (Medline, CINAHL, PsycINFO and Web of Science) and one search engine (Google Scholar). The following keywords were used: *“Patient safety”, “incident reporting”, “Reporting systems”, “learning systems”, “reporting practices”, “Hospital”, “healthcare”* and *“maternity”*. The full search strategy is detailed in online supplemental appendix 1. The first 10 pages of the Google Scholar results were screened due to the broad scope of search engines. The search results were imported into Rayyan https://new.rayyan.ai/^12^ for screening. Duplicates were removed, and forward and backward reference citations of included articles were screened to identify further relevant articles.

### Study selection

Two authors (EB and GB) independently screened titles and abstracts using Rayyan, followed by full-text screening. The authors were blinded to each other’s screening, and any conflicts were resolved by discussion with a third author (CV). The following inclusion criteria were applied: (1) articles published in peer-reviewed journals; (2) studies focusing on patient safety incident reporting practices in maternity care and (3) studies published in English. No restrictions were applied to the year of publication.

### Data extraction

Data were extracted using a prespecified spreadsheet (Excel V.16.5, Microsoft, Redmond WA, USA) with key information included: (1) article characteristics including study design and aims; (2) demographics, staff role and clinical area and (3) outcomes, including the type of incident reporting systems used (eg, name and type), method of incident reporting by healthcare staff (further details on which staff can report the incidents, access to systems), types of incidents reported (eg, how incidents are categorised and which types are most common), barriers to current reporting of incidents and recommendations for improvement. The data extraction fields can be found in online supplemental appendix 2.

### Data synthesis

The extraction form was converted into a Comma-Separated Values (CSV) file and imported into Caplena V2, an Artificial Intelligence (AI) text analysis tool (Caplena AG, Zurich, Switzerland). Caplena was instructed to perform thematic analysis on the key fields relevant to the review research questions including ‘enablers to incident reporting’, ‘types of incidents’, ‘trends in reporting identified in the study’ and ‘recommendations made to improve incident reporting practices’. For each field (eg, ‘Types of incidents’), Caplena coded each data cell and generated themes. One author (EB) manually checked the themes and subthemes and refined some of the themes and subtheme labels by combining, adding or deleting themes. For the first review outcome ‘Methods of reporting incidents’, for example, Caplena initially generated one main theme (‘Reporting methods’) and four subthemes (‘Not applicable’, ‘Checklist’, ‘Paper form’ and ‘Incident reporting system’ (IRS)). These were substantially revised by the first researcher (EB), who reviewed all study data related to this outcome. This included some of Caplena’s predefined subthemes and introduced new codes while refining existing ones. Specifically, the theme ‘Reporting methods’ was changed to ’Reporting system’ and three new subthemes were introduced (‘electronic reporting system’, ‘escalated for review’ and ‘Feedback/action included’). The subthemes ‘paper’ and ‘checklist’ were combined, and ‘IRS/not applicable’ was removed. Additionally, a new theme (‘Reporting requirements’) was created, with subthemes including ‘anonymity’, ‘mandatory/proactive’ and ‘Through senior staff member/risk lead’.

To ensure reliability, the first author went through and coded all data for this outcome using the adapted themes. A second researcher (GB) independently reviewed the coding for this outcome, cross-checked all codes and proposed suggested revisions. These suggestions were discussed with the first author, and changes were made by consensus. A further discussion was held between the two researchers and changes were made including, for example, changing the subtheme of ‘escalated for review’ to ‘Senior staff escalation of incident’. As the machine adapted to prior changes, the need for further adjustments to refine Caplena’s developed themes and subthemes quickly decreased. For the final three review outcomes—‘Enablers’, ‘Gaps’ and ‘Recommendations’, only a few subthemes required adjustments. The automated coding of the data by Caplena also needed minimal refinement.

The remaining review outcomes were analysed in the same structured manner, running Caplena to initially thematically code the data from all the studies per outcome. The first researcher (EB) checked the codes, adapted the themes and subthemes, and added themes where needed. The second researcher then cross-checked all codes, discrepancies were discussed between the two researchers, resulting in changes to the coding. To further enhance reliability, a third researcher (CV) cross-checked 20% of coding and theme/subtheme labelling. CV agreed with all coding and labelling and made no changes.

The resulting codebook is presented in table 1.

**Table 1:** Overview of incident reporting themes and subthemes

Review outcomes	Theme	Subtheme	References
Methods of reporting incidents	Reporting system	Electronic reporting system	^15 17 18 21 23 28^
		Paper report or checklist	^16 17 19 27^
		Feedback/action included	^15 28^
		Senior staff escalation of the incident	^23 28^
	Reporting requirements	Anonymity	^23^
		Mandatory/proactive reporting	^15 19 21^
		Reporting to a senior staff member is required	^16 17 19^
Types of incidents	Clinical	General clinical complications, including blood loss and infection	2126 28
		Procedural errors linked to clinical equipment, procedures or in the lab	^21 27 28^
	Outcome-based incidents	Near misses	^23^
		Level of harm	^23 26^
	Systemic/individual	Communication issues	^23^
		Individual errors	^17 24 26^
		Systemic issues	^24^
Frequency of incident reporting practices	Reported at delivery or frequency over time	Reported incidents at delivery	^29^
		Reporting over time	Did not report change in frequency,^21 27^ increased reporting over time^15^ and decreased over time^28^
Trends in reporting identified in the study	Process/system	Feedback	^15 23 28^
		Differences across clinicians	^21 23^
		Near miss	^15 21 23^
		Clinical errors	^20 21 27^
		Senior staff escalation of the incident	^16 25^
	Culture	Culture of reporting	15 1719 22 26 28
		Communication	^17 23^
		Group consensus	^17^
		Learning from reports	^23^
	Attitudes	Attitudes	1922 25 27
Differences between staff (midwives and obstetricians)	Reporting	Reporting likelihood	15 1827 28
		Reporting process	^23 25^
		Types of incidents reported	^15 18 21 28^
		No differences found	^20^
	Staff	Junior staff higher reporting	^16 18 27^
		Senior staff higher reporting	^17^
		Attitudes to reporting	^17 25^
Barriers to reporting	System/process	Lack of feedback	^22 23^
		Increased workload	^18 24^
		Concerns about the system	^22 24 27^
	Education/experience	Education needed	^21^
		Lack of experience	^18 21 24 27^
	Culture	Unencouraging culture	^21 22^
		Fear of consequences	15 1822 24
Enablers to reporting incidents	System/process	Ease of reporting	^16 21 23 26 27^
		Feedback provided	^15 22 23^
		Anonymity	^17^
		Standardised system	^16^
	Culture	Encouraging culture	1519 22 24 26
		Focus on hazards/near misses	^21 22 24^
		No fear of consequences	1721 23 27
	Education	Education/training provided	^19 23 24 27^
Gaps in reporting processes	System/process	Decision-making in reporting	^20^
		Near misses	^17 21 23^
		Exploration of detail/standardisation in reporting	^17 21 23^
		Inconsistent practices	^17 21^
	Culture	Differences in reporting across clinical settings	^21 23^
		Reporting requirements unclear	^17^
	Education/learning	Education on reporting needs	^23^
		Weak feedback and organisational learning	^16 17 23 28^
Recommendations made to improve reporting processes	System/process	Ease of reporting	^15 17 22 23^
		Research into system improvements	^15 18 28 29^
		Feedback provided	^18 23^
		Research into the analysis of incident data	^15 18 28 29^
	Education	Education/training required	^17 23 25 27 28^
	Culture	Culture change (fostering an open environment)	^16 18 22 23^
		Focus on hazards/near-misses (prevention)	^21 27^
		Good leadership	^23^
		Reflection (encouraged around individual reporting)	^25^

This refinement process highlights the dynamic between algorithmic pattern recognition and human interpretive judgement. Caplena’s initial coding offered a useful foundation by identifying high-frequency terms and provisional categories across datasets. However, in the ‘Methods of reporting incidents’ outcome, many automated outputs reflected surface-level groupings that required contextual interpretation. For instance, the subthemes ‘Checklist’ and ‘Paper form’ were presented as distinct, though manual review revealed they overlapped within paper-based reporting workflows and were therefore combined. Likewise, the subtheme ‘Not applicable’, despite its statistical prominence, was deemed thematically irrelevant and removed. These adjustments went beyond semantics, enabling the identification of more meaningful themes that encompassed broader systemic elements—such as accountability (‘Senior staff escalation of incident’) and infrastructure (‘Electronic reporting system’). This iterative, machine-assisted thematic analysis approach improved coherence and ensured the final coding structure aligned with the review objectives.

### Quality assessment

The quality of included studies was assessed using the Mixed Methods Appraisal Tool (MMAT)^13 14^ as the review included quantitative, qualitative and mixed-methods research designs (see table 2). Two authors (EB and GB) independently rated the articles and resolved any discrepancies through discussion.

**Table 2:** Characteristics of included studies

First author, country	Primary aim of the study	Method/design	Sample and setting	MMAT score
1. Anderson *et al*^20^ USA	To shed light on some factors that may help explain problems with medical error reporting (such as inaccuracy) and that do not receive much attention: ob-gyns’ comfort with reporting medical errors and personal experience with being injured while receiving medical care.	Quantitative descriptive Questionnaire	Obs/gyns=319 Obs/Gyn in primary care and specialist	3.5/5–70%
2. Beigi *et al*^27^ Iran	To identify the status of medical error reporting and related factors among two groups: midwives and midwifery students. Specifically, the study sought to examine the frequency and types of errors reported by midwives and midwifery students. Assess the level of awareness and attitudes towards medical error reporting in both groups. Identify factors related to error reporting behaviour, including the relationship between awareness, attitudes and actual reporting practices. Compare the error reporting practices, awareness and attitudes between practising midwives and midwifery students. Explore potential reasons for differences in reporting practices between the two groups.	Quantitative descriptive—correlational study Questionnaire	Midwives=86 Midwifery students=100 All midwifery and labour departments in the hospital and university for the students	4.5/5–90%
3. Currie *et al*^21^ USA	To describe the frequencies and types of hazard and near-miss events reported across three cohorts of BS/MS students who used the web-based system in the first year of their combined BS/MS Advanced Practice Nurse programme.	Longitudinal observational design—mixed methods Collection and analysis of hazard and near-miss reports	Students=453 Obstetrics in hospital	2.5/5–50%
4. Hewitt *et al*^23^ Canada	To understand the different stages of electronic incident reporting and to do so in a comparative study of two hospital divisions: General Internal Medicine, Obstetrics and Neonatology.	Qualitative—comparative case study Interview	General medicine, obstetrics and neonatology HCPs=85 Hospital	5/5–100%
5. Howell *et al*^15^ UK	To examine whether annual hospital incident reporting rates can be used as a surrogate indicator of individual hospital safety. Secondly, it assesses which hospital characteristics are correlated with high incident reporting rates and whether a high reporting hospital is safer than those with lower reporting hospitals. Finally, it assesses which healthcare professionals report more incidents of patient harm, which report more near-miss incidents and what hospital factors encourage reporting.	Quantitative descriptive Incident data analysis and correlation with hospital characteristics and staff survey results	Incident reports from 148 trusts=5 879 954 All clinical areas	5/5–100%
6. Jager *et al*^28^ Switzerland	(1) To determine the distribution of critical incidents across clinical specialities, (2) to describe CIRS reporter’s professional profiles, (3) to explore types, severity and risk of reoccurrence of critical incidents and (4) to investigate factors contributing to such incidents and preventive actions that have been taken in response.	Quantitative retrospective, cross-sectional study Analysis of incident reports	Critical incident cases=5493 All clinical areas	4/5–80%
7. Lawton and Parker^16^ UK	Investigates the willingness of healthcare professionals (doctors, nurses and midwives) to report colleagues to a superior member of staff following an adverse incident or near miss and explores the difference in reporting of events involving three kinds of behaviour distinguished by Reason *et al*—compliance with a protocol, violation of a protocol and improvisation where no protocol exists.	Quantitative retrospective, cross-sectional study Questionnaire	HCPs=315 All clinical areas	3.5/5–70%
8. Lindsay *et al*^17^ UK	To obtain a picture of the social and cultural influences on reporting behaviour in the maternity services of a large inner city National Health Service hospital Trust.	Qualitative-ethnographic approach Participant observation and interviews	Maternity staff=32 Delivery unit, antenatal, postnatal ward and community	5/5–100%
9. Majda *et al*^25^ Poland	The purpose of this study was to measure the attitudes of nurses working in internal medicine departments and surgical departments, as well as midwives working in obstetrics departments, towards adverse events. In addition, the relationship of attitudes towards clinical adverse events with selected sociodemographic variables, such as seniority, age and education, is analysed.	Cross-sectional survey Survey	Nurses of internal medicine wards=547 Nurses of internal surgical wards=168 Midwives=120 Internal medicine and surgical departments, and obstetrics departments	4/5–80%
10. Majda *et al*^26^ Poland	To learn about the experiences of clinical adverse events by nurses working in internal medicine and surgical wards and midwives, as well as their involvement in the recognition and reporting of such events, including their perceptions.	Cross-sectional Questionnaire	Internal medicine wards=457 Surgical wards=168 Maternity wards=120	4/5–80%
11. Twijnstra *et al*^29^ Netherlands	To evaluate the feasibility of such a complication registration system for obstetricians/gynaecologists. Second, the validity, comprehensiveness and completeness of the registration system were evaluated. Finally, the differences in relative frequencies of complications reported when using the new registration system between the participating hospitals in this pilot study were analysed.	Prospective observational multicentre design (quantitative) Standardised form used to register all complications, during admission and up to 6 weeks after discharge	Complications observed=351 Obstetrics and Gynaecology	3/5–60%
12. Vincent *et al*^18^ UK	To explore the reasons for low incident reporting rates with the aim of refining and improving existing systems.	Quantitative descriptive Questionnaire	Midwives=98.1% Doctors=84% Obstetrics	3/5–60%
13. Waring^19^ UK	This paper describes the intrahospital variations in incident reporting within five specialist medical departments and gives an account of the associated attitudes and practices of physicians.	Observational Overt observations in the management structure of a hospital. F2F interviews with managers and clinicians across the hospital	Direct involvement in management and admin of quality improvement, risk management or incident reporting=12 Consultants in five departments=25 Risk leaders=4 Obstetrics, including other departments	2/5–40%
14. Waters *et al*^24^ Canada	To explore Canadian labour and delivery nurses’ perceptions about reporting incidents in practice and identify factors facilitating or constraining incident reporting.	Qualitative descriptive Focus groups, interviews	Nurses on labour and delivery units=16 Labour and delivery units	5/5–100%
15. Zabari and Southern^22^ USA	To understand how the experiences of shame and guilt, coupled with organisational factors, affect error reporting by obstetric clinicians. The purpose of our study was to bring a systems perspective to the individual and organisational factors that facilitate or create barriers to error reporting among obstetric clinicians.	Descriptive cross-sectional. Interviews (semistructured)	Nurse-midwife=67 Physician=17 Maternity units	3/5–60%

CIRS, Critical Incident Reporting System; F2F, face to face; HCP, healthcare professional; MMAT, Mixed Methods Appraisal Tool; Obs/gyn, Obstetrics/Gynaecology.

### Patient and public involvement (PPI)

The PPI team (JD, RK and KG) have been involved in the review since the inception of the wider workstream on maternity incident reporting practices in October 2023. They did not have input into the research questions or design of the review. All three members provided feedback on the first draft of the review, and revisions were based on their feedback. The PPI team will be involved in disseminating the findings through PPI groups and social media. They will also be involved in helping shape the interview study that follows on from this systematic review.

## Results

### Study characteristics

A total of 15 studies met the inclusion criteria. These studies were conducted in seven countries: five in the UK,^15^,^19^ three in the USA,^20^,^22^ two in Canada,^23 24^ two in Poland^25 26^ and one each in Iran,^27^ Switzerland^28^ and the Netherlands.^29^ The studies employed a diverse range of methodologies, including quantitative surveys, qualitative interviews and mixed methods designs. Descriptive and cross-sectional designs were used to explore reporting behaviours and practices among healthcare professionals.^1620 25^,^27^ Longitudinal, observational and prospective designs examined incident tracking systems and critical incident data across clinical specialities.^21 28 29^ Lastly, qualitative methods such as interviews, participant observation and focus groups explored the social, cultural and emotional factors influencing reporting practices within healthcare environments.^1722^,^24^

Seven studies examined the reporting behaviour and experiences among specific healthcare professionals (midwives, midwifery students, nurses or obstetricians) focusing on factors influencing their reporting practices^18 20 21 24 26 27^ or to evaluate the incident reporting methods themselves.^29^ Two additional studies explored adverse events, investigating factors that shape healthcare professionals’ willingness to report incidents involving colleagues and their attitude towards adverse events.^22 25^

In contrast to studies that focused on particular groups within the healthcare system, five studies examined incident reporting at the systemic level.^15 16 19 23 28^ Howell *et al*^15^ examined incident reporting practices across 148 hospital trusts, while Lawton and Parker^16^ explored incident reporting across three hospital trusts. Hewitt *et al*^23^ investigated electronic incident reporting across two hospital divisions, and Waring^19^ and Jäger *et al*^28^ analysed hospital-wide incident reporting rates and characteristics across one hospital. Additionally, Lindsay *et al*^17^ and Waring^19^ explored the social and cultural influences on hospital reporting structures. Lindsay *et al*^17^ focused on maternity services, whereas Waring^19^ examined variations in reporting practices across five medical departments. An overview of the characteristics of the 15 included studies is presented in table 2.

### Current incident reporting practices in maternity care: systems used

**Figure 2:**
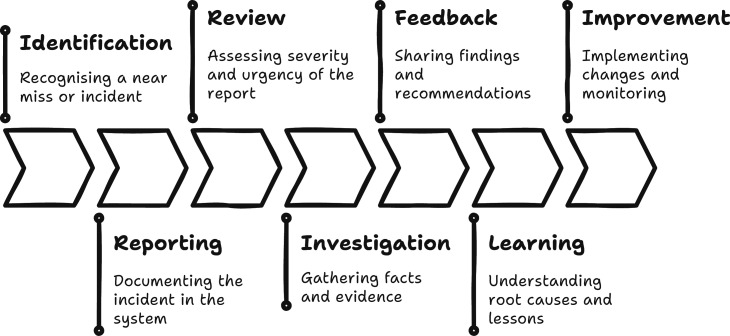
The incident reporting cycle.

Three studies reported the use of non-electronic reporting systems, such as paper incident forms/Trust intranet/electronic records,^17^ complication registration forms^29^ or researcher developed checklists to document and understand incidents.^27^ In contrast, five studies reported using an electronic reporting system, though some did not specify which system was used. Three studies explicitly named the reporting systems used. These were the National Reporting and Learning System (NRLS) in the UK,^15^ the Medical Event Reporting System for Hospitals (MERS-TH) in the USA^21^ and the Critical Incident Reporting System medical in Switzerland.^28^ The studies highlighted key features of the reporting system, such as the ability to provide feedback to the reporting staff member and the option to escalate a report to senior staff. These features enhanced communication and accountability in incident management. The remaining seven studies did not mention a reporting system. A simplified conceptual model of the incident reporting cycle is presented in figure 2, outlining the sequential stages through which safety incidents are identified, reported, investigated and translated into organisational learning and improvement.

### How do incident reporting practices differ in maternity care?

Incident reporting practices in maternity care varied across studies, particularly in how incidents were categorised. Most studies classified incidents based on clinical factors, grouping them into subcategories such as blood loss, infection or type of clinical issues.^1821 26^,^29^ Other studies adopted an outcomes-based approach, categorising incidents by the level of harm caused or distinguishing between incidents, hazards and near misses.^23 24 26^ Additionally, some studies categorised incidents into systemic or individual errors, identifying whether the issue stemmed from organisational processes or personal actions.^17 23 24 26^ This distinction is critical for understanding whether errors stem from workplace systems, staffing issues or procedural inefficiencies, rather than solely attributing blame to individuals.

### Barriers and enablers to reporting incidents

Barriers to incident reporting were categorised into three main areas: organisational culture, workload/time pressures and limitations of existing reporting platforms. Organisational culture played a significant role in staff members’ willingness to report patient safety incidents.^21^ For instance, Howell *et al*^15^ indicated that fear of penalties in some organisations deterred reporting. The fear of consequences and the lack of a learning and no-blame culture were the main reasons why staff did not report incidents.^17 18 22 24^ Hewitt *et al*^23^ emphasised that organisations without a learning culture failed to provide feedback to staff after an incident was reported, further discouraging staff from reporting. One nurse reported that a systems view would help to learn from incidents:

For me it’s very helpful because now I can see trends… (People) individually have their own problem, but this now allows us to see it as a systems issue. So we notice that this mistake is happening with this medication or this process so we can go back and discuss it. We are able to pinpoint a systems issue rather than reflect on one individual issue, which for me is very helpful because it’s education, it’s global, it’s not a problem with a nurse, it’s usually related to a system.^23^

A high workload and time pressures acted as barriers to the reporting of incidents,^18 24^ along with patient safety reporting systems not being considered fit for purpose.^24 27^

Conversely, several enablers were identified to improve incident reporting. Training and raising awareness on the benefits of incident reporting were key enablers that encouraged staff engagement.^23^ Strong leadership support from hospital leadership was also highlighted as a factor that fostered a supportive hospital culture, characterised by non-punitive environments and learning from incidents.^22^,^24^ In Zabari and Southern’s study, one clinician explained the need for a non-punitive environment to feel safe enough to report incidents:

I bet you (that) the people making mistakes could tell a lot if they felt safe to do so and (could) be part of the solution. If I knew my manager wasn’t going to, you know, give me a 2 on my eval(uation), if I knew my peers weren’t going to talk behind my back when I leave my shift. I might want to report.^22^

**Figure 3:**
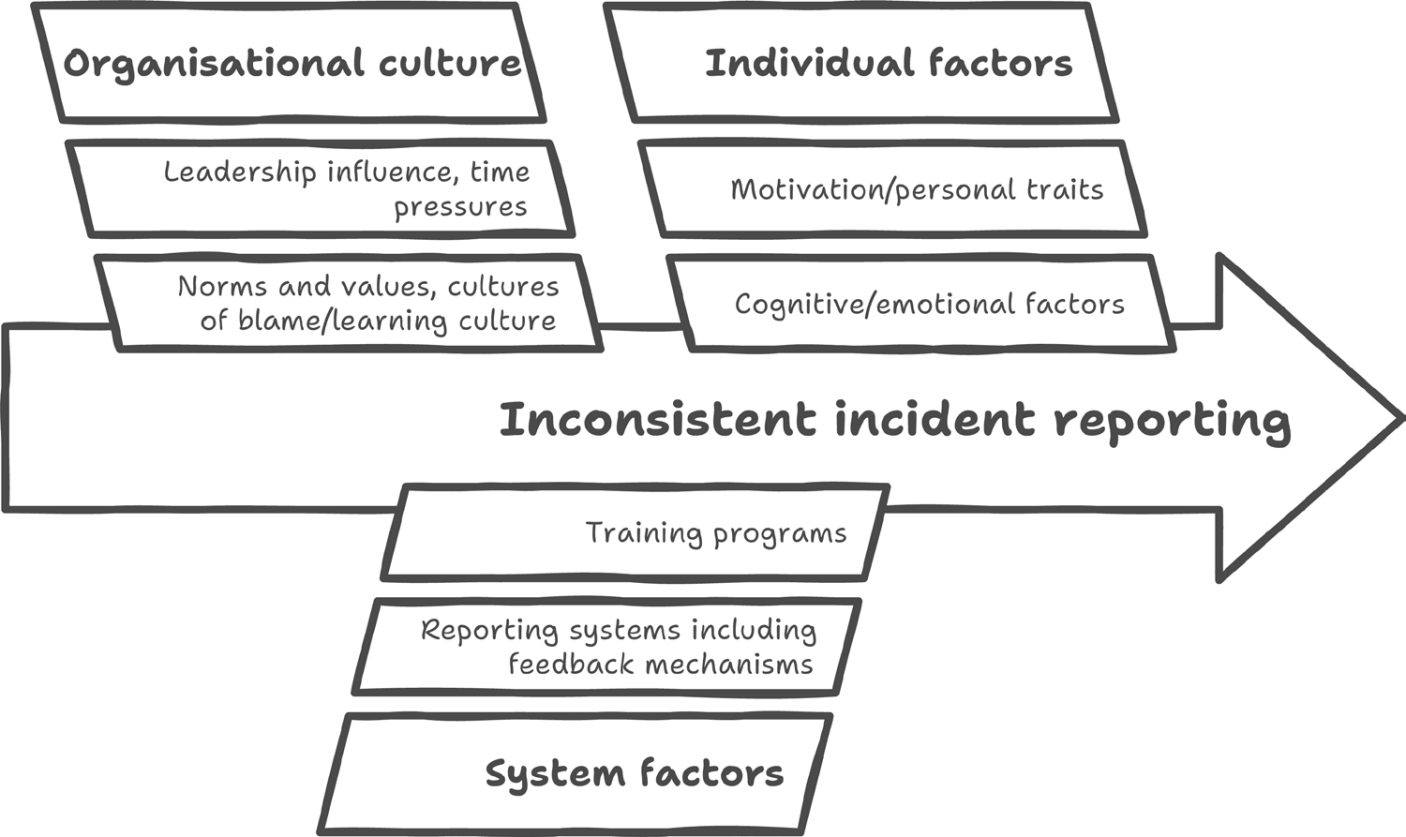
Incident reporting behaviours influence model.

The process of learning included the integration of feedback mechanisms after reporting.^15 23^ Well-designed reporting platforms were also identified as enablers, especially those that could be designed to facilitate reporting, and staff could easily access,^23^ used standardised templates^16^ and allowed qualitative data in the form of comments on the report.^21^ The incident reporting behaviours influence model outlines the key facilitators and barriers that affect an individual’s willingness to report safety incidents (see figure 3).

### Current gaps in patient safety incident reporting practices

Several key gaps in patient safety incident reporting were identified across the studies. A major issue was the disconnect between incident reports and organisational learning, particularly the lack of feedback loops after incidents were reported.^28^ Another gap was the lack of exploration of staff experiences with the incident and reporting process.^17^ Several authors mentioned the under-reporting of near misses and variation in reporting practices across different settings.^17 21 23^ The exertion of managerial power in incident reporting was mentioned by one midwife in Lindsey *et al*’s ethnographic study:

…and apparently her incident form went in the bin. And so she came back saying, ‘Would you believe my incident form was binned!’^17^

Howell *et al*^15^ also identified the lack of ethnicity data in reports, which limits the ability to analyse potential biases.

### Recommendations for improving patient safety incident reporting

The main recommendations made across the articles focused on the importance of creating a supportive, non-punitive environment, with leadership strongly committed to patient safety.^23^ Other recommendations included training on patient safety reporting,^17 28^ implementing a reporting system fit for purpose (described as simple and standardised),^18^ and enabling the integration of free text in reports to fully describe incidents.^15^

See table 1 for an overview of the themes and subthemes identified in the studies.

## Discussion

### Key findings

The findings of this review highlight the differences in incident reporting practices within maternity care settings. The review found that although electronic systems are primarily used for incident reporting, their effectiveness is often hindered by punitive workplace cultures, workload pressures and inadequate systems.^17 18 23^

An organisational culture that penalises staff for reporting incidents was a major deterrent to reporting.^15 21^ The fear of negative consequences, compounded by a lack of feedback and organisational learning, discouraged staff from engaging in the reporting process.^17 18 22 24^ A qualitative study in obstetrics^30^ found that education and simulation training were important in reducing the likelihood of making errors and improving patient safety culture. The study emphasised that clearly defined processes for handling errors offer midwives protection and security. Additionally, the pressure of workload and time constraints was frequently cited as a barrier to effective reporting.^18 24^ These findings suggest that the culture of safety within healthcare settings plays a crucial role in either fostering or hindering reporting practice. However, these approaches must be tailored to the organisation in terms of relevance and tangible benefits,^31^ which requires further research.

In contrast, several enablers to incident reporting were identified to facilitate incident reporting, including organisational support, leadership commitment and streamlined reporting systems. Training and raising awareness about the benefits of reporting, as well as creating a supportive, non-punitive environment, emerged as key factors facilitating reporting.^23^ Increasing awareness of incident reporting can positively influence healthcare professionals’ attitudes and behaviours, reducing uncertainty around when and how to report incidents. Strong leadership was highlighted as instrumental in cultivating a culture of safety. When leaders demonstrated a commitment to patient safety, staff were more likely to report incidents.^22 23^ This aligns with findings from Pedroni *et al*,^32^ which suggest that errors should be viewed as systemic issues within the organisational system rather than the result of isolated professional actions. Furthermore, the design of reporting platforms also played a significant role. Systems that were user-friendly, standardised and capable of collecting qualitative data encouraged transparent and better reporting practices.^23^ However, it is concerning that even in high-income countries with national universal healthcare systems, few reporting platforms incorporate qualitative data in the reporting of patient safety incidents.^33^

Our review further indicates that incident reporting is not solely a function of technological systems or external organisational policies but is also deeply influenced by entrenched professional hierarchies and moral experiences. Hierarchical structures can intensify a culture of fear and blame, where individuals may experience profound shame or even the ‘second victim’ phenomenon when errors occur.^34^,^36^ This moral burden can deter open reporting and exacerbate feelings of isolation among healthcare professionals, suggesting that efforts to improve incident reporting must also address these emotional and ethical dimensions. As such, fostering psychological safety through supportive leadership, debriefing and targeted training may help alleviate these adverse effects and encourage a more transparent and compassionate reporting culture.

The review also identified varying trends in incident reporting practices across studies. Although only five studies directly assessed the frequency of reporting incidents, their findings suggest a lack of consistency in how incident reporting is measured and reported. Some studies showed an increase in reporting rates over time, while others observed a decrease. This discrepancy may be attributed to differences in reporting systems, the nature of the incidents recorded or levels of staff engagement. Additionally, inconsistencies in how incidents are categorised, whether based on clinical factors, outcomes or systemic issues, further complicate comparisons across studies. These variations reflect the complexity of understanding and reporting incidents in maternity care. It is important to consider whether higher rates of reporting, particularly of near misses, contribute to improved safety outcomes. A system that encourages the reporting of near misses alongside actual incidents can provide valuable insights into potential risks, allowing organisations to proactively mitigate harm rather than reacting to adverse events after they occur. Moreover, as AI is being increasingly integrated into healthcare, its ability to analyse and learn from incident reports can enhance the efficiency and effectiveness of reporting systems, ensuring that patterns and trends are identified and addressed before they lead to harm. Despite the insights provided by the reviewed studies, several gaps in the current literature were identified. The absence of a feedback loop and the disconnect between incident reports and organisational learning could undermine the effectiveness of reporting systems.^28^ Additionally, the under-reporting of near misses and the variation in reporting practices across different settings remain significant concerns.^17 21 23^ Furthermore, the lack of inclusion of ethnicity data in reports^15^ limited the ability to assess and address potential biases in reporting practices. These gaps underline the need for further research to better understand how incident reporting systems can be improved to encourage more comprehensive and accurate reporting.

Studies from the UK, USA and Switzerland pointed to electronic reporting systems with a particular focus on the NRLS in the UK and MERS-TH in the USA. Since its implementation in 2001, the NRLS has collected over 20 million incident reports.^37^ However, the full potential of this information had not been fully used, leading to the development of a new Patient Safety Incident Management System (PSIMS) to address these shortcomings. Introduced in 2021, PSIMS aims to improve reporting capabilities by automating uploads, making data more accessible, and providing better feedback to staff and organisations.^37^

Learning from well-established incident reporting systems in other industries offers valuable insights into enhancing safety and accountability in healthcare. The Aviation Safety Reporting System, managed by the National Aeronautics and Space Administration, provides a confidential mechanism for aviation professionals to report safety concerns without fear of punishment.^38^ This open culture of reporting has contributed to critical improvements in air travel, such as enhanced cockpit communication protocols and proactive hazard identification. Similarly, the European Rail Agency has developed a structured incident reporting system across railway networks, facilitating the identification of recurring safety risks and leading to standardised safety measures that reduce accidents.^39^ Adopting principles from these systems, such as encouraging confidential reporting, ensuring a non-punitive approach and implementing standardised procedures, can strengthen healthcare incident reporting. By fostering a culture where staff feel safe to report issues, organisations can better identify patterns, address systemic challenges and ultimately improve patient safety.

While our synthesis is not explicitly framed within a particular theoretical framework, it aligns closely with the principles of sociotechnical systems theory, safety-II and human factors theory. Our analysis acknowledges the interplay between organisational culture, leadership and reporting system functionality, key elements of a sociotechnical perspective. The identification of barriers such as under-reporting and lack of feedback reflects the need for a holistic approach that integrates human, technological and organisational factors to enhance patient safety. Additionally, our emphasis on fostering a supportive, non-punitive culture resonates with safety-II principles, which advocate strengthening adaptive capacities rather than solely focusing on failures. Furthermore, our work aligns with human factors theory by recognising the impact of cognitive workload, communication and system usability on incident reporting behaviours. By integrating these perspectives, our synthesis provides valuable insights into improving reporting practices in maternity care without being constrained by a singular theoretical lens.

Regarding the methodology, Caplena was chosen over a purely manual thematic analysis because it effectively addresses several challenges inherent in manual coding, namely, the process is labour-intensive, time-consuming and prone to subjective inconsistencies. By using AI-driven text analysis, Caplena provided an objective, rapid initial coding of diverse study data, establishing consistent preliminary themes that could then be refined by researchers. There are lessons to be learnt from using an AI tool, Caplena, to aid the thematic analysis of the review extraction data. Caplena proved particularly beneficial in two key ways:

Theme transferability: when prompted by Caplena during each analysis with the question ‘Start from scratch?’, the team could select no, allowing previously coded themes and subthemes from related outcomes to be transferred across (if appropriate) to other outcomes, such as those from Barriers to be transferred into Enablers. This enabled more precise thematic categorisation of data within each outcome.

Machine learning adaptation: as themes were refined, Caplena incorporated researcher-led adjustments and learnt from modifications made to previous coding. This iterative training process reduced the need for exhaustive manual coding, as the platform adapted to prior decisions, requiring only verification rather than full recoding of all data. Over time, Caplena’s learning curve resulted in fewer necessary adjustments, increasing efficiency.

Although the scope of data in this study was relatively limited, yielding only marginal reductions in workload, Caplena’s functionality presents significant potential for larger-scale reviews. Its ability to streamline thematic analysis, minimise coding duplication and facilitate systematic data management enhances research efficiency. Additionally, Caplena offers sentiment analysis capabilities alongside thematic analysis, providing further analytical depth where required.

### Limitations of the review

This review has some limitations. First, despite employing a comprehensive search strategy using a librarian, the restriction of databases may have led to the omission of relevant studies. However, we made every effort to at least identify all literature within the constraints of these databases. Second, the language restrictions may have led to a selection bias, as studies published in other languages were excluded, thereby narrowing the diversity of perspectives and findings considered. The findings may lack generalisability due to being context-specific or influenced by particular population characteristics, limiting their applicability to broader or more diverse groups. Finally, inherent biases within the included studies, such as methodological flaws or sampling issues, could impact the overall quality and validity of the findings. To account for this, we have formally assessed study quality using MMAT so that findings can be considered in context. We have clearly described the study contexts, allowing readers to make informed inferences with regard to generalisability.

### Recommendations for future research and practice

The reviewed studies provided several recommendations for improving patient safety incident reporting in maternity care. A supportive, non-punitive environment that fosters trust and encourages incident reporting without fear of retribution is essential. This involves leadership commitment to safety, training on reporting benefits and developing user-friendly, standardised reporting systems that capture both quantitative and qualitative data.^15 18^ Future research should evaluate the effectiveness of these recommendations across various healthcare settings and consider cultural and contextual factors affecting reporting practices. A major gap in current reporting systems is the lack of ethnicity data, despite evidence showing higher rates of adverse outcomes in non-White groups.^40^,^42^ This omission significantly limits the ability to analyse disparities and implement targeted safety improvements. Given the findings from studies such as Farrant *et al*’s retrospective review on ethnicity and serious incidents in maternity care,^43^ incident reporting systems must incorporate ethnicity data to ensure its impact on maternal outcomes is properly understood and addressed. Additionally, research must examine the impact of feedback mechanisms on reporting rates, ensuring that healthcare professionals receive meaningful responses and system-wide improvements following incident reports. Future studies should also account for the hospital context, such as rural versus urban settings, resource availability, as well as whether there is sufficient expertise to translate and adapt learning effectively.^44^ In practice, we propose several targeted features and interventions for future incident reporting systems. First, designing a user-centred interface that incorporates real-time feedback dashboards, an emphasis on near-miss reporting and the ability to submit reports anonymously. This approach helps reduce the fear of retribution, break down hierarchical barriers and shift the focus from assigning blame for incidents to learning from near misses, ultimately fostering a culture of safety and improvement. Second, integration of AI-driven analytics and dashboards may enable early detection of incident trends and facilitate proactive system improvements, while embedding structured peer-support and debriefing modules can help address the moral and emotional burdens (eg, the second victim phenomenon) experienced by staff. To operationalise these strategies, future systems could include mobile-enabled, voice-to-text reporting tools that allow clinicians to submit incidents in real time, even during busy shifts. Anonymous two-way messaging features would enable reporters to receive updates or clarifications without compromising confidentiality. Integrated peer-support prompts could be triggered after high-impact incidents, offering staff immediate access to trained colleagues or well-being resources. Additionally, systems could incorporate automated reminders for follow-up actions, ensuring that learning from incidents is not lost over time. Finally, linking incident data with staffing levels or patient acuity scores could provide a richer context for understanding contributory factors and inform more effective system-level responses. These specific strategies provide a roadmap for enhancing transparency, fostering a learning culture and ultimately improving patient safety outcomes in maternity care.

## Conclusion

This review highlights the complex landscape of incident reporting in maternity care, where factors such as reporting system design, organisational culture and workload pressures play critical roles in shaping reporting practices. Addressing the factors that act as barriers and enablers in reporting offers significant potential to improve incident reporting and enhance patient safety. However, further research is needed to explore the nuances of reporting systems, particularly in relation to feedback, near misses and the inclusion of demographic data, particularly ethnicity. There is a clear call to action for stakeholders in maternity care to prioritise patient safety incident reporting. In the UK, the introduction of the PSIRF offers maternity units a unique opportunity to reassess and strengthen their incident reporting structures. By embracing the principles of proactive learning, meaningful feedback loops and structured risk analysis, maternity units can move beyond passive incident logging towards an approach that actively informs safety interventions. To facilitate progress, stakeholders should implement clear strategies for engaging staff in incident reporting, integrate near-miss reporting to mitigate future harm and ensure robust analysis of incidents to drive system-wide learning.

## Supplementary material

10.1136/bmjoq-2025-003432 online supplemental appendix 1

10.1136/bmjoq-2025-003432 online supplemental appendix 2

## Data Availability

All data relevant to the study are included in the article or uploaded as supplementary information.
