# Supplementary material for: Systematic review of patient safety incident reporting practices in maternity care
Source: BMJ Open Qual. 2025 Oct 5;14(4):e003432. doi: 10.1136/bmjoq-2025-003432 (PMC12506158; doi:10.1136/bmjoq-2025-003432)
Supplement: online supplemental appendix 1 [file bmjoq-14-4-s001.docx]

**Appendix 1- Medline search strategy**

Ovid MEDLINE(R) ALL <1946 to June 25, 2024>

1 Midwif*.ti,ab.

2 Midwifery/

3 maternal health services/ or exp perinatal care/ or prenatal care/

4 (perinatal or prenatal or peri-natal or pre-natal).ti,ab.

5 Pregnancy/ or Pregnancy Complications/

6 Obstetrics/

7 (pregnan* or obstetric* or maternit* or maternal or labor or labour or childbirth* or child-birth* or antenatal* or ante-natal* or antepartum or ante-partum or prenatal* or pre-natal*).ti,ab.

8 error*.ti,ab.

9 adverse event*.ti,ab.

10 safety.ti,ab.

11 medical errors/ or near miss, healthcare/

12 Accident Prevention/

13 Safety/ or Safety Management/

14 Medical Errors/

15 Medication Errors/

16 Organizational Culture/

17 Risk Management/

18 risk management.ti,ab.

19 safety management,.ti,ab.

20 ((reporting or reported) adj3 (system or systems or database* or practice* or process* or incident or incidents)).ti,ab.

21 Learning system*.ti,ab.

22 (detect$ adj (error$ or incident$ or accident$)).ti,ab.

23 (Reporting adj (error$ or incident$ or accident$)).ti,ab.

24 Information Systems/

25 Incident$ report$.ti,ab.

26 reporting culture.ti,ab.

27 1 or 2 or 3 or 4 or 5 or 6 or 7

28 8 or 9 or 10 or 11 or 12 or 13 or 14 or 15 or 16 or 17 or 18 or 19

29 20 or 21 or 22 or 23 or 24 or 25 or 26

30 27 and 28 and 29
