# Supplementary material for: Systematic review of patient safety incident reporting practices in maternity care
Source: BMJ Open Qual. 2025 Oct 5;14(4):e003432. doi: 10.1136/bmjoq-2025-003432 (PMC12506158; doi:10.1136/bmjoq-2025-003432)
Supplement: online supplemental appendix 2 [file bmjoq-14-4-s002.docx]

**Appendix 2- Data extraction fields**

| Category | Field name |
| --- | --- |
| Article characteristics | Title |
|  | Author |
|  | Year of publication |
|  | Country of study |
|  | Document type |
|  | Aims |
|  | Study design |
|  | Methods |
|  | Analysis method |
| Demographic data | Healthcare staff (role) |
|  | Maternity only/Hospital-wide |
|  | Clinical population |
|  | Clinical area (antenatal ward/community midwifery |
|  | Sample size (staff) |
|  | Age range |
|  | Gender |
|  | Actual reporting practices or perceived / hypothetical reporting practices |
| Outcomes | Details of reporting system/s in study (name and type) |
|  | Methods of reporting incidents |
|  | Types of incidents |
|  | Severity of incidents (near misses/incidents-no harm-severe harm) |
|  | Frequency of reporting incidents |
|  | Trends in reporting identified in the study |
|  | Differences between staff (midwives vs obstetricians) |
|  | Barriers to reporting incidents |
|  | Enablers to reporting incidents |
|  | Gaps in reporting practices |
|  | Recommendations made to improve reporting practices |
|  | Limitations of the study as identified by the authors |
| Other | Other useful findings |
|  | Additional comments |
